# Fast Generation of Simulation-Quality Structural Ensembles of Mixed-Chirality Cyclic Peptides via Diffusion Models

**DOI:** 10.1021/acs.jctc.5c01862

**Published:** 2026-03-12

**Authors:** Nomindari Bayaraa, Maxim Secor, Marc L. Descoteaux, Yu-Shan Lin

**Affiliations:** Department of Chemistry, 1810 Tufts University, Medford, Massachusetts 02155, United States

## Abstract

Cyclic peptides are an emerging therapeutic modality, with recent computational efforts focusing on the design of cyclic peptides that predominantly adopt a single conformation. However, many cyclic peptides adopt multiple conformations in solution, existing as structural ensembles. This conformational flexibility is often integral to their function: chameleonic switching between alternative states can enhance membrane permeability, and specific conformations may be required for molecular recognition and binding. Consequently, the ability to predict their structural ensembles is crucial for advancing the de novo design of cyclic peptide therapeutics. Here, we introduce diffusion models to efficiently and accurately predict structural ensembles of mixed-chirality cyclic peptides. The models are trained directly on molecular dynamics (MD) simulation data; in particular, each frame of the simulation becomes a single training instance in which a structure is represented as sine and cosine values of backbone dihedral angles. The trained diffusion model can not only generate MD-quality structures of cyclic peptides, but also the generated structures follow the Boltzmann distribution sampled in the MD simulation, enabling a deeper understanding of the physicochemical basis of cyclic peptide properties and allowing efficient computational design of cyclic peptides targeting biologically relevant systems.

## Graphical abstract

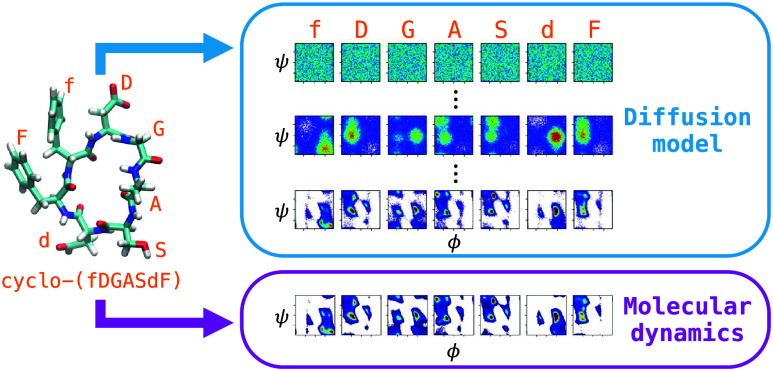

## Introduction

Numerous vital biological processes, such as cell-to-cell interactions and signal transductions, are regulated by protein–protein interactions (PPIs).^1^ Due to the large, shallow interaction surfaces involved in the PPIs, small molecules are often too small to modulate PPIs.^2–4^ On the other hand, biologics can target large surfaces involved in PPIs, but these molecules typically exhibit poor cell membrane permeability.^5,6^ Cyclic peptides have emerged as a promising drug modality because they can exhibit desirable properties of small molecules and biologics concurrently, as evidenced by their ability to permeate cell membranes and modulate PPIs.^4,5,7,8^ Moreover, cyclic peptides exhibit enhanced stability, specificity, and improved resistance to proteolytic degradation compared to their linear counterpart.^9–11^ However, the development of cyclic peptide drugs—especially, de novo design—is hindered by limited structural information.

Despite continuous advances in experimental techniques, characterizing cyclic peptide structures remains challenging. For instance, the characterization of cyclic peptides using solution NMR is difficult because cyclic peptides tend to adopt multiple conformations in solution, existing as a structural ensemble.^12–14^ Conversely, computational methods can provide the necessary information, making techniques like molecular dynamics (MD) simulation an attractive alternative.^11,15,16^ Unfortunately, MD simulation is both computationally expensive and time-consuming, rendering it unsuitable for large-scale screening.

Progress in deep learning approaches for the prediction of cyclic peptide structures has been hindered by limited structural information and the resulting scarcity of adequately sized training data. Despite the challenges, several methods have been introduced in recent years. AfCycDesign^17^ and the HighFold series^18–20^ are AlphaFold2-derived models that incorporate cyclic relative positional encoding to predict cyclic peptide structures. CyclicBoltz1 ^21^ incorporates another version of cyclic relative positional encoding suited for Boltz1,^22^ an open source model achieving AlphaFold3-accuracy. RFpeptide^23^ also uses cyclic relative positional encoding for RFdiffusion^24^ to generate cyclic peptide binders against protein targets. These methods all modify pretrained protein models that were trained on the Protein Data Bank,^25^ which means the training data set is heavily biased toward homochiral cyclic peptides, namely, l-amino acids and Gly. Further, the trained models do not capture structural ensembles. Alternatively, Rosetta^26^ and CyclicCAE^27^ can sample heterochiral cyclic peptide backbones. Rosetta uses physics-based energy functions and a generalized kinematic closure algorithm for conformational sampling. CyclicCAE, an autoencoder trained on a Rosetta-generated data set, allows more efficient sampling than Rosetta. However, both Rosetta and CyclicCAE are limited to low-energy backbone conformations and require subsequent sequence design with Rosetta. Most notably, RINGER^28^ is a diffusion-based generative model that can generate sequence-conditioned structural ensembles of cyclic peptides with 4, 5, and 6 amino acids. However, RINGER is trained on the Conformer-Rotamer Ensembles of Macrocyclic Peptides (CREMP) data set,^29^ which comprises structural ensembles of macrocyclic peptides in chloroform, generated using the Conformer-Rotamer Ensemble Sampling Tool (CREST).^30^ CREST employs direct conformational sampling at a semiempirical quantum chemical level to produce conformer ensembles. Furthermore, our previous work, Structural Ensembles Achieved by Molecular Dynamics and Machine Learning (StrEAMM) models, can predict structural ensembles of cyclic pentapeptides^31^ and cyclic hexapeptides^32^ in water. These StrEAMM models are trained on MD simulation data and employ structural digit maps to discretize and define structural ensembles of cyclic peptides observed in explicit water simulations. However, the models are not natively extensible to other cyclic peptide ring sizes and residues due to the use of a structural digit map tailored for each scaffold.

In this work, we introduce diffusion models that enable rapid and accurate generation of structural ensembles of cyclic pentapeptides, cyclic hexapeptides, and cyclic heptapeptides (Figure 1). We employ a denoising diffusion probabilistic model (DDPM),^33^ which is a type of deep generative model that tries to learn the underlying probability distribution of data by slowly and iteratively noising data in a forward process and then restoring the data with a reverse process (Figure 1C). At each step of the reverse process, a U-Net^34^ predicts the amount of noise to be removed (Figure 1B). The diffusion models for cyclic peptides are trained directly on MD simulation data, allowing the models to generate MD-quality structures of cyclic peptides. In particular, we convert each frame of MD simulation into a single training instance by extracting backbone dihedral angles (ϕ, ψ) of the cyclic peptide structure and representing the structure as a matrix containing sine and cosine values of the (ϕ, ψ) angles. For instance, one frame from the MD simulation of cyclo-(fDGASdF), where lowercase letters represent d-amino acids, will turn into a 7 × 4 matrix where each row corresponds to one of the seven residues, and the four columns contain the sin ϕ, cos ϕ, sin ψ, cos ψ information of the residues, respectively (Figure 1A). Each training instance is paired with a 7 × 15 matrix of sequence encoding, where each row represents one-hot encoding of a residue (Figure 1A). Importantly, sequence-conditioning allows the generated structural ensembles to follow the Boltzmann distribution observed in the MD simulation of a given cyclic peptide. The trained diffusion models can accelerate the resource-intensive drug discovery process by allowing efficient query of the cyclic peptide sequences and screening based on their ability to preorganize to a desired conformation.

**Figure 1:**
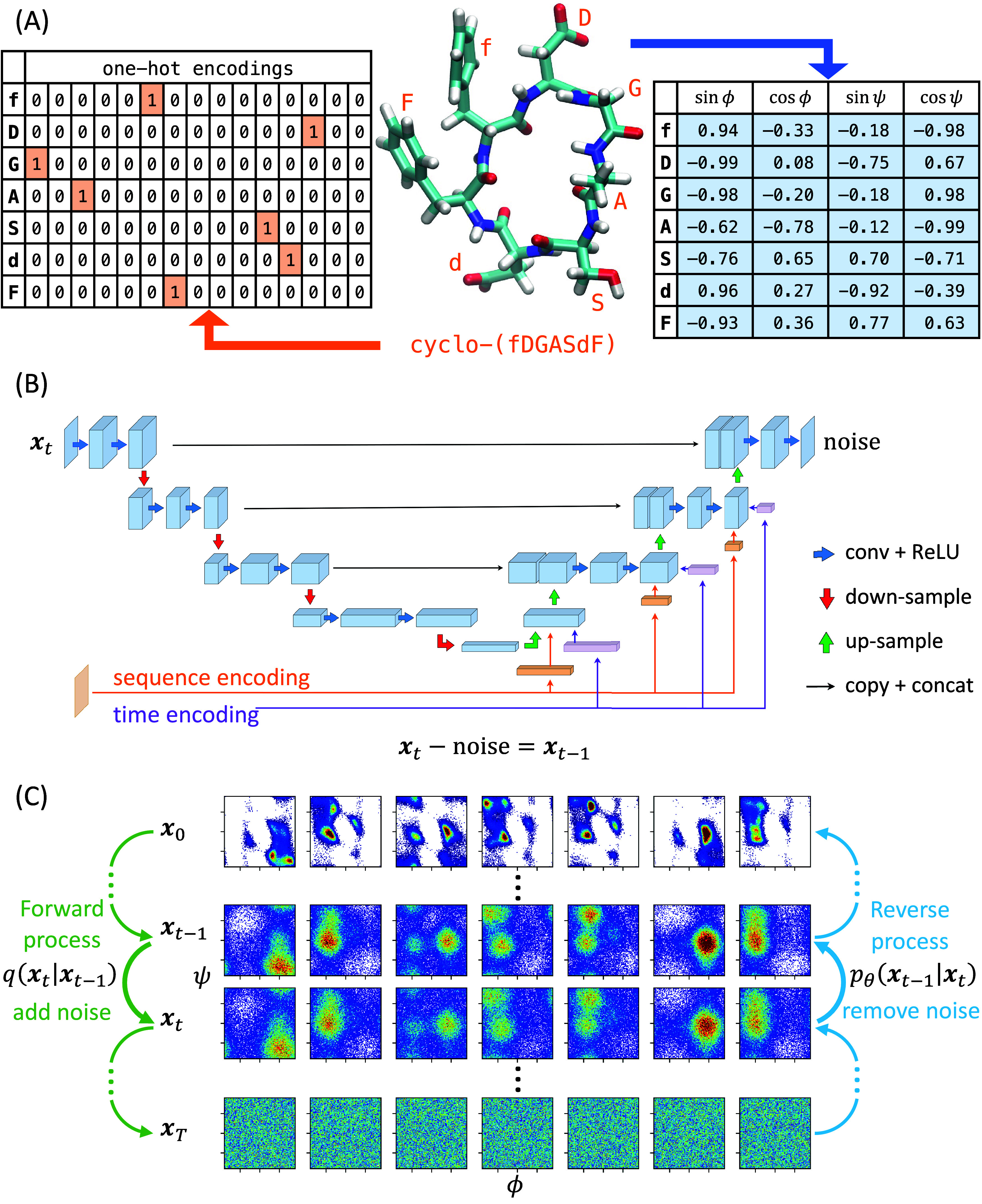
Overview of the diffusion models. (A) Each structure observed in a molecular dynamics simulation is converted into a pair of matrices representing the sequence and structure of a cyclic peptide. The orange matrix on the left contains sequence information, where each row contains the one-hot encoding of a residue. The blue matrix on the right is a single training instance representing a structure, where each row contains the sine and cosine transformation of the backbone dihedral angles (ϕ, ψ) of a residue. (B) A variant of the U-Net architecture is implemented in this work to predict the noise to be removed during the reverse process. At each step, U-Net takes a noisy 7 × 4 structure matrix (***x***_*t*_) along with the corresponding sequence encoding (in orange) and time encoding (in purple) and outputs a “noise matrix” which is then subtracted from the noisy matrix ***x***_*t*_ to obtain a less noisy matrix ***x***_*t*–1_. (C) During the model training, a known amount of noise is gradually added to the noise-free matrix ***x***_0_ until it becomes pure noise ***x***_*T*_. When making an inference, sequence-conditioned noise is gradually removed from a random pure noise ***x***_*T*_ to generate a structure matrix, ***x***_0_.

## Methods

### MD Simulations of Cyclic Heptapeptides

We curated a data set for cyclic heptapeptides by generating a list of cyclic heptapeptide sequences and performing MD simulations of these sequences. Similar to our previous works on StrEAMM linear regression models^31^ and StrEAMM neural network models,^32^ these cyclic heptapeptide sequences were generated from a library of 15 amino acids: G, A, V, F, N, S, D, R, a, v, f, n, s, d, and r, where the lowercase letters represent the d-amino acids. This set of 15 amino acids was chosen to provide a diverse and representative subset of the canonical amino acids along with their d forms. G is achiral and the smallest amino acid, while A is the simplest chiral amino acid. V contains a β-branched side chain, F an aromatic ring, N an amide group, S a hydroxyl group, D a negatively charged side chain, and R a positively charged side chain.

The training sequences were generated with two different protocols: semirandom and random. The semirandom protocol efficiently generates a set of sequences that ensures all possible *Χ_i_Χ_i_*_+1_ *Χ*_*i*+2_ subsequences (where *Χ* is one of the 15 amino acids) are observed at least once. The random protocol is a straightforward generation of random cyclic heptapeptide sequences using the library of 15 amino acids. The semirandom protocol resulted in 502 cyclic heptapeptide sequences (see List S1). An additional 300 sequences were generated with the random protocol (List S2). Another 53 sequences were generated with the random protocol as test sequences (List S3), and these test sequences do not overlap with the 802 training sequences.

Cyclic heptapeptides in the training and test set sequences were simulated, following a MD simulation protocol similar to our previously published works.^31,32^ All MD simulations were performed with GROMACS 2018.6 ^35^ patched with PLUMED 2.5.1 plugin^36^ using the RSFF2 force field^37^ with the TIP3P water model.^38^ For each cyclic heptapeptide, we performed two independent bias-exchange metadynamics (BE-META) simulations starting from two distinct initial structures to assess the simulation convergence.^39–41^ The two initial structures were prepared with the UCSF Chimera package.^42^ In order to ensure the two initial structures were sufficiently different, we enforced a criterion requiring the backbone root-mean-square deviation (RMSD) between the two structures to be at least 2.0 Å. The prepared initial structures were energy minimized in vacuum using the steepest descent algorithm. Then the energy-minimized cyclic peptide was solvated in a box of pre-equilibrated water molecules, ensuring that the minimum distance between the peptide and the walls of the box was at least 1.0 nm. When the total charge of the cyclic peptide was nonzero, minimal simple counterions (Na^+^ or Cl^–^) were added to neutralize the system. The solvated system was energy-minimized once again with the steepest descent algorithm.

After the energy minimization, a four-step equilibration process was conducted before a production run. First, a 50 ps NVT simulation at 300 K was conducted during which the heavy atoms of the cyclic peptide were positionally restrained by a harmonic potential with a force constant of 1,000 kJ mol^–1^ nm^–2^. Second, a 50 ps NPT simulation at 300 K and 1 bar was conducted while maintaining the same positional restraints as the first step. Third, a 100 ps NVT simulation at 300 K was conducted, but all positional restraints were removed to allow the whole system to equilibrate. Lastly, a 100 ps NPT simulation at 300 K and 1 bar was conducted without any positional restraint.

The production NPT BE-META simulation was carried out for up to 400 ns at 300 K and 1 bar. Each production simulation had a total of 19 replicas: seven replicas biasing the 2D collective variables (ϕ*_i_*, ψ_*i*_), seven replicas biasing the 2D collective variables (ϕ*_i_*, ψ_*i*‑1_),^41^ and five neutral replicas without bias. The Leapfrog algorithm with a time step of 2 fs was used for dynamics evolution. A V-rescale thermostat^43^ was used for temperature control, regulating the cyclic peptide and solvent separately, each with a coupling time constant of 0.1 ps. A Parrinello–Rahman barostat^44^ was used for pressure control with a coupling time constant of 2 ps and an isothermal compressibility of 4.5 × 10^–5^ bar^–1^. During equilibration, all bonds were constrained with the LINCS algorithm;^45,46^ during production, bonds involving hydrogen were constrained with the LINCS algorithm. Electrostatic and van der Waals interactions were truncated at 1.0 nm. Long-range electrostatics were treated using the particle mesh Ewald summation^47,48^ with a Fourier grid spacing of 0.12 nm and an order of 4. A long-range dispersion correction was applied to correct energy and pressure.

To monitor the simulation convergence, we conducted dihedral principal component analysis (dPCA)—a variant of PCA in which conformations are described by backbone dihedral angles (ϕ, ψ) represented by sine and cosine transformations—to yield a rotation- and translation-invariant low-dimensional representation.^49^ We then projected backbone dihedral angles onto the top three principal components and calculated the resulting 3D probability density for the two independent sets of simulations for each cyclic peptide sequence. An overlap between the two densities *P*_1_(*r*) and *P*_2_(*r*) from the two independent simulations was quantified with the normalized integrated product (NIP)^50^

$$\mathrm{NIP}=\frac{2\int P_{1}\left(r\right)P_{2}\left(r\right)dr}{\int P_{1}^{2}\left(r\right)dr+\int P_{2}^{2}\left(r\right)dr}$$

The NIP value ranges from 0 (distributions without any overlap) to 1 (identical distributions). If the NIP between the two independent simulations was ≥0.9, we considered the simulations to be converged. For both the training and test data sets, all simulations were initially performed for 100 ns. Using the last 50 ns of the five neutral replicas, if the NIP between the density distributions of the two independent simulations was ≥0.9, we considered the simulations converged (Figure 2, orange). If the NIP value was <0.9, the simulations were extended for another 100 ns (Figure 2, green). Then, the last 100 ns of the five neutral replicas were used to calculate the NIP. If the NIP was still <0.9, then the simulations were extended for another 100 ns, meaning the total production run at this point would be 300 ns (Figure 2, blue). Here, the last 100 ns of the simulations (200–300 ns) were initially used to calculate the NIP. If the resulting NIP was <0.9, then the last 200 ns of the simulations (100–300 ns) were used to determine convergence. If the simulations were not converged, the simulations were extended for another 100 ns (Figure 2, purple). Similarly, the last 100, 200, and 300 ns of the simulations were used to determine convergence. If the simulations were not converged even after 400 ns, we discarded the sequence from the data set to ensure only high-quality data were included. The final training data set contained simulations of 734 sequences: 467 semirandom sequences (Figure 2A) and 267 random sequences (Figure 2B). The final test set contained simulations of 50 random sequences (Figure 2C), none of which overlapped with the 734 sequences in the training set.

**Figure 2:**
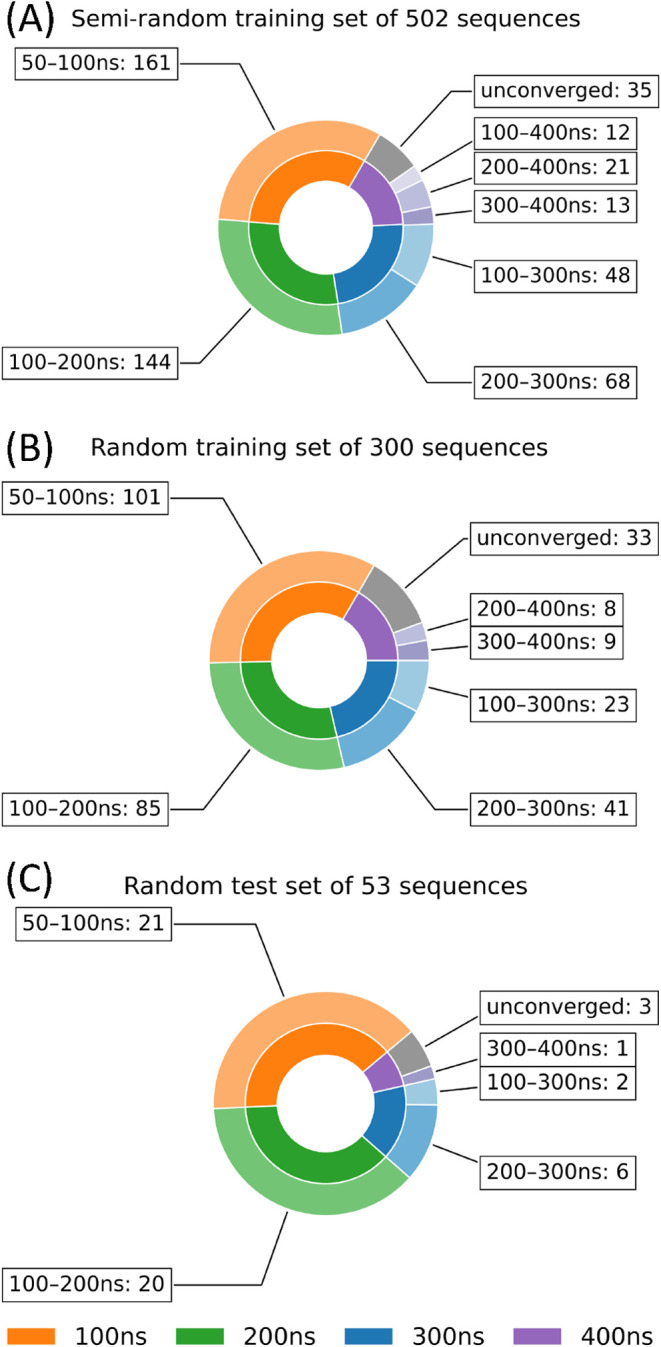
Convergence status of the cyclic heptapeptide simulations. (A) Semirandom training data set contains 467 converged simulations of cyclic heptapeptide sequences where all possible *X*_*i*_ *X*_*i*+1_ *X*_*i*+2_ subsequences are observed at least once (*X* is one of the 15 amino acids). Among the 502 sequences, simulations of 467 converged within 400 ns, and the remaining 35 were discarded from the data set to ensure high-quality data. (B) Random training data set. Among the 300 sequences, simulations of 267 converged within 400 ns, and the remaining 33 sequences were discarded. (C) Random test data set. None has any overlap with (A, B). Among 53 sequences, simulations of 50 converged within 400 ns, and the remaining 3 sequences were discarded.

### Construction of Data Sets for Diffusion Models

We processed the simulation data of all three systems (cyclic pentapeptide, cyclic hexapeptide, and cyclic heptapeptide) and encoded the sequences and structures as matrices (Figure 1A). To allow for a fair comparison with our previously published StrEAMM models for cyclic pentapeptides^31^ and cyclic hexapeptides,^32^ we used the simulations of the same training and test sequences. For cyclic pentapeptides, a training data set of 705 sequences and a test data set of 50 sequences were utilized in this work, which were derived from a 15-amino-acid library as described in our previously published work on the StrEAMM model for cyclic pentapeptides.^31^ For cyclic hexapeptides, a training data set of 705 sequences and a test data set of 49 sequences were used, which were also derived from the same 15-amino-acid library as described in our previous work.^32,54^ For cyclic heptapeptides, a training data set of 734 sequences and a test set of 50 sequences were used as described in the previous section. The diffusion models for cyclic pentapeptides, hexapeptides, and heptapeptides are trained independently, and the models do not share any layers or parameters.

While training a model on the entire simulation data is desirable, the large size of the simulations—often comprising millions of frames—poses considerable computational challenges. To mitigate this challenge, we aimed to identify an optimal subsampling that reduces the data volume while maintaining the essential conformational coverage. Multiple versions of the training and test data sets were generated by subsampling the simulation trajectories to generate 1k, 5k, 10k, 25k, 50k, and 75k frames per sequence. To evaluate the coverage retained in each subset, the subsampled trajectories were compared to the full trajectories using dPCA analysis, followed by NIP calculation, consistent with the process used to assess convergence of two simulations. As shown in Figure 3, the similarity between the full trajectory and the subsampled trajectories starts to plateau at 25k frames. Hence, we used the training and test data sets containing 50k frames per sequence. Then we characterized the cyclic peptide conformations contained in each simulation frame using a matrix representation.

**Figure 3:**
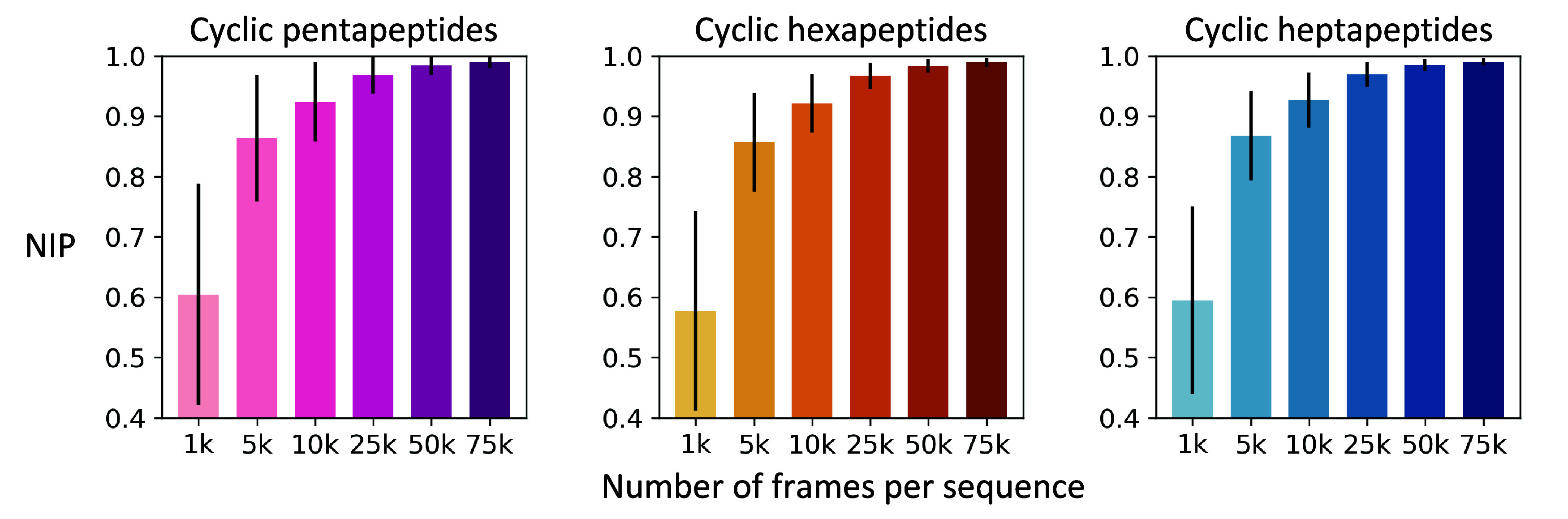
To identify an optimal subsampling that reduces the data volume while maintaining essential conformational coverage, we subsampled the simulation trajectories to generate 1k, 5k, 10k, 25k, 50k, and 75k frames per sequence. The similarity between the full trajectory and the subsampled trajectories was quantified with the normalized integrated product (NIP) value between the distributions of backbone dihedral angles projected onto 3D principal component space, as described in the Methods section. The similarity between the full trajectory and the subsampled trajectories begins to level off at around 25k frames per sequence. Therefore, the diffusion models were trained on the data set with 50k frames per sequence.

In particular, we converted each frame of the subsampled trajectory into a single training instance by extracting backbone dihedral angles (ϕ, ψ) of a cyclic peptide conformation and representing the conformation as a matrix containing the sine and cosine values of ϕ, ψ angles. For instance, one frame from the MD simulation of cyclo-(fDGASdF) is converted into a 7 × 4 matrix where each row contains the sin ϕ, cos ϕ, sin ψ, cos ψ values of a residue, as shown in Figure 1A. With the goal of achieving sequence-conditioned conformation generation, each training instance is paired with a 7 × 15 matrix representation of the sequence, where each row represents the one-hot encoding of a residue. Because the cyclic peptide sequences used in this work are generated from a 15-amino-acid library, the one-hot encoding of a residue is a bit vector of length 15. One bit vector contains a single value of “1” at a position unique for each of the 15 amino acids in the library, and the rest of the vector has a value of “0.” While one-hot encoding provides a simple way to represent amino acids and is straightforward to implement, it does not capture the physicochemical properties of amino acids.^51^ We note that more descriptive encodings could potentially enhance the performance of the diffusion models.^51–54^

To encourage the models to be properly cyclically permuted, meaning a cyclic permutation of an input sequence leads to a corresponding cyclic permutation of the output structure, we enriched the data set with cyclic permutations of each sequence. For example, cyclo-(fDGASdF) has 50k subsampled frames from its simulation. Then its six cyclic equivariant sequences—cyclo-(DGASdFf), cyclo-(GASdFfD), cyclo-(ASdFfDG), cyclo-(SdFfDGA), cyclo-(dFfDGAS), and cyclo-(FfDGASd)—were added to the data sets, with both the sequence matrix and the structure matrix permutated accordingly. As a result, another 300k frames corresponding to the cyclic equivariant sequences were added to the data set. Also, we exploited the enantiomeric nature of amino acids by further enriching the data set with enantiomers (mirror images) of the sequences. For example, cyclo-(FdGasDf) is an enantiomer of cyclo-(fDGASdF); so, the structure matrix of cyclo-(FdGasDf) was constructed to reflect the sign inversion of ϕ, ψ, and the sequence matrix was updated to reflect conversions between the selected subset of l- and d-amino acids. Hence, the final data sets contain both the cyclic permutations of each sequence and their enantiomers, resulting in a total of 734 × 14 = 10,276 cyclic heptapeptide training sequences, for example.

### Conditional Diffusion Models

We utilized a denoising diffusion probabilistic model (DDPM),^33^ which is a type of deep generative model that gradually perturbs data by adding noise and learns to reconstruct the original data by removing the added noise.^55^ DDPM typically incorporates two Markov chains: a forward process and a reverse process.

The forward process gradually adds Gaussian noise to the original data, effectively transforming it into a latent representation ***x***_*t*_ over discrete time steps

t=1,...,T

At each time step, the noise is introduced according to a fixed Markov process

$$q\left(x_{t}|x_{t-1}\right)=N\left(x_{t};\sqrt{1-\beta_{t}}x_{t-1},\beta_{t}Ι\right)$$

Here, β_*t*_ determines the variance of the added noise, and β_*t*_ ∈ (0,1] following the relationship 0 < β_1_ < β_2_ <...< β*_T_* < 1 defines the noise schedule. **I** is the identity matrix, and N(x;μ,Σ) denotes the multivariate normal probability density function evaluated at ***x*** with mean **μ** and covariance **Σ**. The full forward process can be described as a Markov chain conditioned on the input data ***x***:

$$q\left(x_{1:T}|x_{0}\right)=\prod_{t=1}^{T}q\left(x_{t}|x_{t-1}\right)$$

This process converts ***x***_0_ into pure Gaussian noise $x_{T}∼N\left(0,I\right)$ . If α_*t*_ = 1 – β_*t*_ and α̅_*t*_ = ∏_*s*=1_^*t*^α_*s*_, the distribution of the noisy sample ***x**_t_* at any arbitrary time step *t* can be expressed in closed form

$$q\left(x_{t}|x_{0}\right)=N\left(x_{t};\sqrt{{\mathrm{\alpha ̅}}_{t}}x_{0},{(1-\mathrm{\alpha ̅}}_{t}\right)Ι)$$

which means any ***x***_*t*_ can be obtained directly from the distribution conditioned on the original input ***x***_0_. This reparameterization allows expressing noisy samples as

$$x_{t}=\sqrt{{\mathrm{\alpha ̅}}_{t}}x_{0}+\sqrt{{(1-\mathrm{\alpha ̅}}_{t})}\epsilon$$

where ϵ∼N(0,I) is the injected Gaussian noise. In the original DDPM work,^33^ the authors set the noise scheduler constants to be small relative to their data scaled to [−1,1] and keep a small signal-to-noise ratio (≈10^–5^) at ***x***_*T*_. Because the structures of cyclic peptides are represented as sine and cosine values of backbone dihedral angles (ϕ, ψ), the domain of the data set used in this work is also [−1,1]. Thus, we employed the same noise scheduler as Ho et al^33^

$$T=1000,\;\beta_{1}=10^{-4},\mathrm{and}\;\beta_{T}=0.02$$

The reverse process is modeled as a parametrized Markov chain, where the model is trained to denoise pure Gaussian noise iteratively and restore the original data

$$p_{\theta }\left(x_{0:T}\right)=p\left(x_{T}\right)\prod_{t=1}^{T}p_{\theta }\left(x_{t-1}|x_{t}\right)$$

where $p\left(x_{T}\right)=N\left(x_{T};0,I\right)$ . Each transition is Gaussian

$$p_{\theta }\left(x_{t-1}|x_{t}\right)=N\left(x_{t-1};\mu_{\theta }\left(x_{t},c,t\right),\Sigma_{\theta }\left(x_{t},t\right)\right)$$

where **μ**_θ_(***x***_*t*_ *,**c**,t*) is the predicted mean of the denoised sample, **Σ**_θ_(***x***_*t*_ *,t*) is the variance, and ***c*** is an optional conditioning information. Instead of directly predicting the mean, the neural network is trained to predict the Gaussian noise ***ϵ*** that was added at step *t*, ***ϵ***_*θ*_ (***x***_*t*_ *,**c**,t*), by minimizing the loss: $E_{x_{0},c,t}\left[{∥\epsilon -\epsilon_{\theta }\left(x_{t},c,t\right)∥}^{2}\right]$ .

To improve the conditional generation, we used classifier-free guidance (CFG),^56^ which has been demonstrated to enhance the quality of diffusion-generated samples by combining the unconditional noise estimate, ***ϵ***_θ_(***x***_*t*_ *,t*), with the conditional noise estimate, ***ϵ***_θ_(***x***_*t*_ *,**c**,t*), as

$${\mathrm{\epsilon ̂}}_{\theta }\left(x_{t},c,t\right)=\left(1+\omega \right)\epsilon_{\theta }\left(x_{t},c,t\right)-\omega \epsilon_{\theta }\left(x_{t},t\right)$$

where ω is the guidance strength. During the model training, random dropout of the conditioning is applied to jointly train conditional and unconditional models.

### Protocol for Hyperparameter Tuning and Model Selection

We optimized the diffusion model hyperparameters on the validation sets that were held out from training and never used for final testing (Figure S1). To stabilize the optimization, we always applied an exponential moving average (EMA)^57^ to the model weights, as EMA has been demonstrated to improve model generalization and sample quality. For conditioning inputs, we fixed the dropout rate to be 0.1 during training, both to regularize the network and to improve the robustness in CFG inference. The mini-batch size was held constant at 512 across all trials to isolate the effects of other hyperparameters. We varied the learning rate and architectural parameters—notably, the U-Net channel width (number of features) and depth (number of down-sampling layers)—that directly determine the number of trainable parameters of the model. To mitigate overfitting, we implemented early stopping with a patience of five checkpoints, such that training was terminated if the validation loss did not decrease by at least 0.1% across five consecutive evaluations. We first performed a randomized search to explore broad hyperparameter ranges and then refined promising regions with a grid search. All models were trained to convergence under these criteria, after which we selected the optimal hyperparameter and trained two additional models using different training/validation splits. We report the final performance on the test set using the three independently trained models to account for variability due to data splits and random initialization.

During the hyperparameter tuning, we trained several different model sizes with a number of trainable parameters ranging from 401 thousand to 180 million (Figure S2) and validated the models on one validation split. The inference time scales linearly with the model size (Figure S3A), while the performance shows diminishing returns beyond a model with 50 million trainable parameters (Figure S3B). Consequently, we stopped the hyperparameter search at a channel width of 512 and the model depth of four (Figure S4). For the cyclic pentapeptides, the largest model achieved the best validation performance. For cyclic hexapeptides and cyclic heptapeptides, the performance differences between the largest and the second-largest models on the validation sets were minimal, whereas the inference time nearly doubled. Thus, we selected the largest cyclic pentapeptide model and the second-largest cyclic hexapeptide and heptapeptide models as the final models. The final models were used for subsequent tuning of ω (guidance strength) using the same validation sets. We tested several values of ω and found that the performance increased slightly with an increase in ω up to a certain point, followed by a linear decline as ω continued to increase (Figure S5).

All models were trained using Python 3.8 ^58^ with PyTorch^59^ 2.4 and CUDA 12.0 on eight NVIDIA L40 GPUs. CPU resources consisted of 32 Intel Xeon Gold 6448Y processors running Red Hat Enterprise Linux 8.9. Training times for the final diffusion models, as well as their inference times, are reported in Table S1.

## Results and Discussion

### Diffusion Model Accurately Predicts the Structural Ensembles of Cyclic Pentapeptides

To determine the diffusion model’s ability to recover structural ensembles of cyclic pentapeptides, we generated 50k structures (each represented by a 5 × 4 structure matrix) for each of the 50 test set sequences. The generated structures were compared to those observed in the MD simulations by quantifying their distribution similarity with NIP. Two complementary representations of the conformational space were examined. First, similar to the assessment of simulation convergence, we performed dPCA on both the MD results and the diffusion model results, followed by the calculation of NIP between the MD and diffusion model distributions of backbone dihedral angles projected onto the top three PC space. An example comparison between such distributions for cyclo-(avNSd) is shown in Figure 4A. Further, all cyclic permutations of cyclo-(avNSd) were generated to ensure that the diffusion model can handle cyclic permutations of sequences (Figure S6). Figure 7A shows the probability distribution of the NIP values for all 50 test sequences calculated in the PC space across the three diffusion models (with different training validation splits); the average NIP is 0.948, suggesting that the diffusion models are able to generate structures that follow the structural ensembles observed in MD simulations. Second, we examined the Ramachandran plots, i.e., the 2D distributions of the backbone dihedral angles, to quantify per-residue similarity between the generated and observed structural ensembles. For example, Figure 4B shows the per-residue NIPs between the (ϕ, ψ) distributions of cyclo-(avNSd) observed in the MD simulation (Figure 4B, top) and generated with the diffusion model (Figure 4B, bottom). The per-residue NIP values confirm that the Ramachandran plots of the observed and generated structural ensembles are nearly identical. Figure 7B shows the probability distribution of the NIP values for all 50 test sequences calculated in the Ramachandran space across the three diffusion models; the average NIP is 0.983.

**Figure 4:**
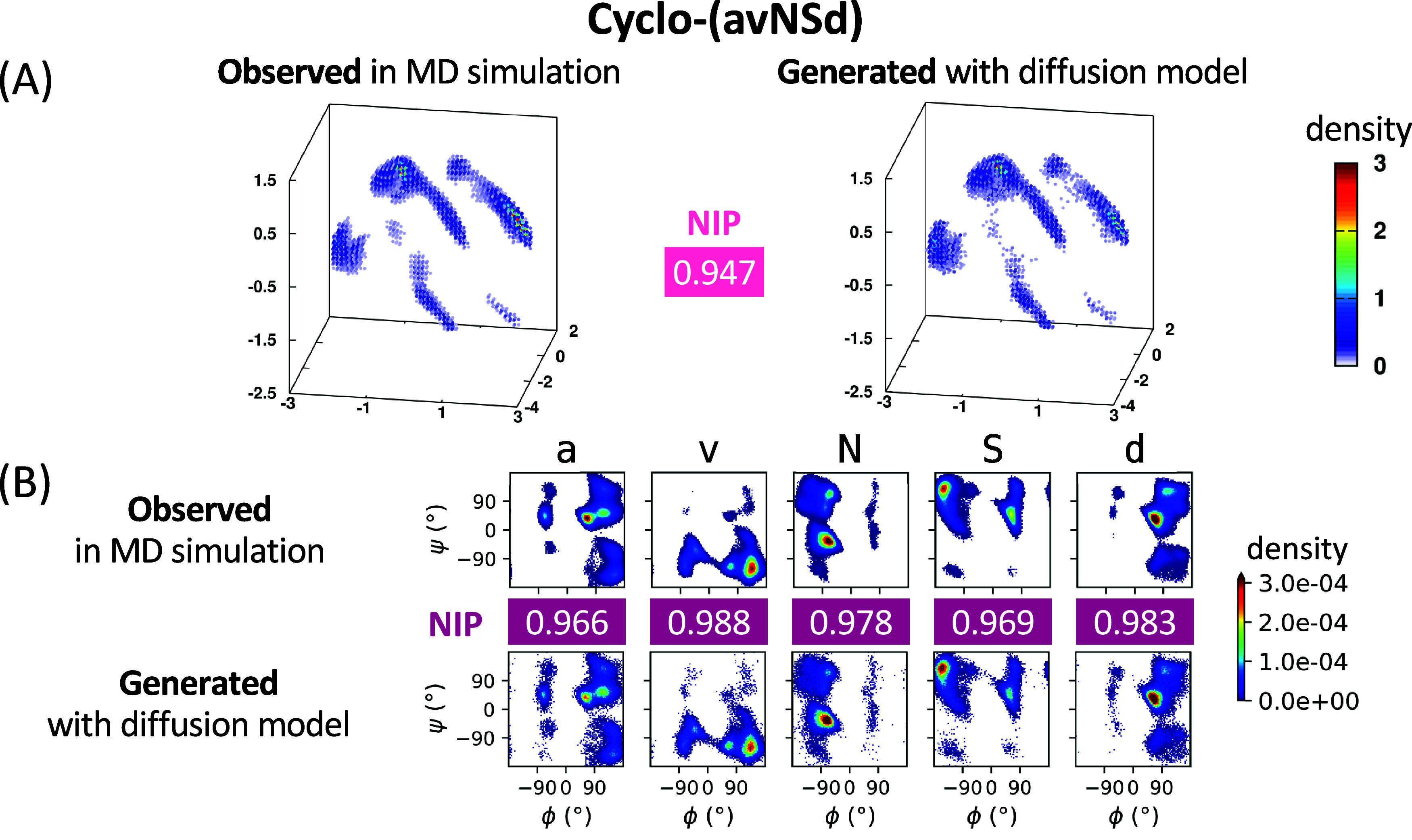
Prediction of the structural ensembles of cyclo-(avNSd). (A) Projections of backbone dihedral angles (ϕ, ψ), expressed as sine and cosine, onto 3D principal component spaces. NIP between the 3D probability distributions of the MD-observed and the diffusion model-generated structural ensembles was 0.947. (B) Ramachandran plots of the MD-observed and the diffusion model-generated structural ensembles. Per-residue NIP between 2D distributions of the backbone dihedral angles (ϕ, ψ) ranged from 0.966 to 0.988.

### Diffusion Model Accurately Predicts the Structural Ensembles of Cyclic Hexapeptides

Similar to what we did to evaluate the model performance for cyclic pentapeptides, we assessed the capacity of the cyclic hexapeptide diffusion model to reproduce the MD structural ensembles by generating 50k structures (each encoded as a 6 × 4 structure matrix) for each of the 49 sequences in the test set. To benchmark against MD simulation, we quantified distributional similarity between the generated and simulated ensembles using NIP. Ensemble comparisons were carried out in two complementary representations of conformational space. In the first analysis, we applied dPCA, projecting sine and cosine transformations of backbone dihedral angles onto the top three PCs and computing the NIP between the resulting distributions (see Figure 5A for cyclo-(RdnDFf) as an example). Figure 7C shows the distribution of the NIP values in the PC space for all test 49 sequences; the average is 0.933, indicating close agreement between generated and MD ensembles. In a second analysis, we examined ensembles by comparing the two-dimensional (ϕ, ψ) distributions on a per-residue basis. For example, cyclo-(RdnDFf) showed highly similar Ramachandran densities between the MD-observed (Figure 5B, top) and the diffusion model-generated structural ensembles (Figure 5B, bottom), as reflected in their high per-residue NIP values. When averaged across the entire test set, the Ramachandran analysis produced an average NIP of 0.966, further confirming that the model reliably recapitulates the conformational distributions observed in MD (Figure 7D).

**Figure 5:**
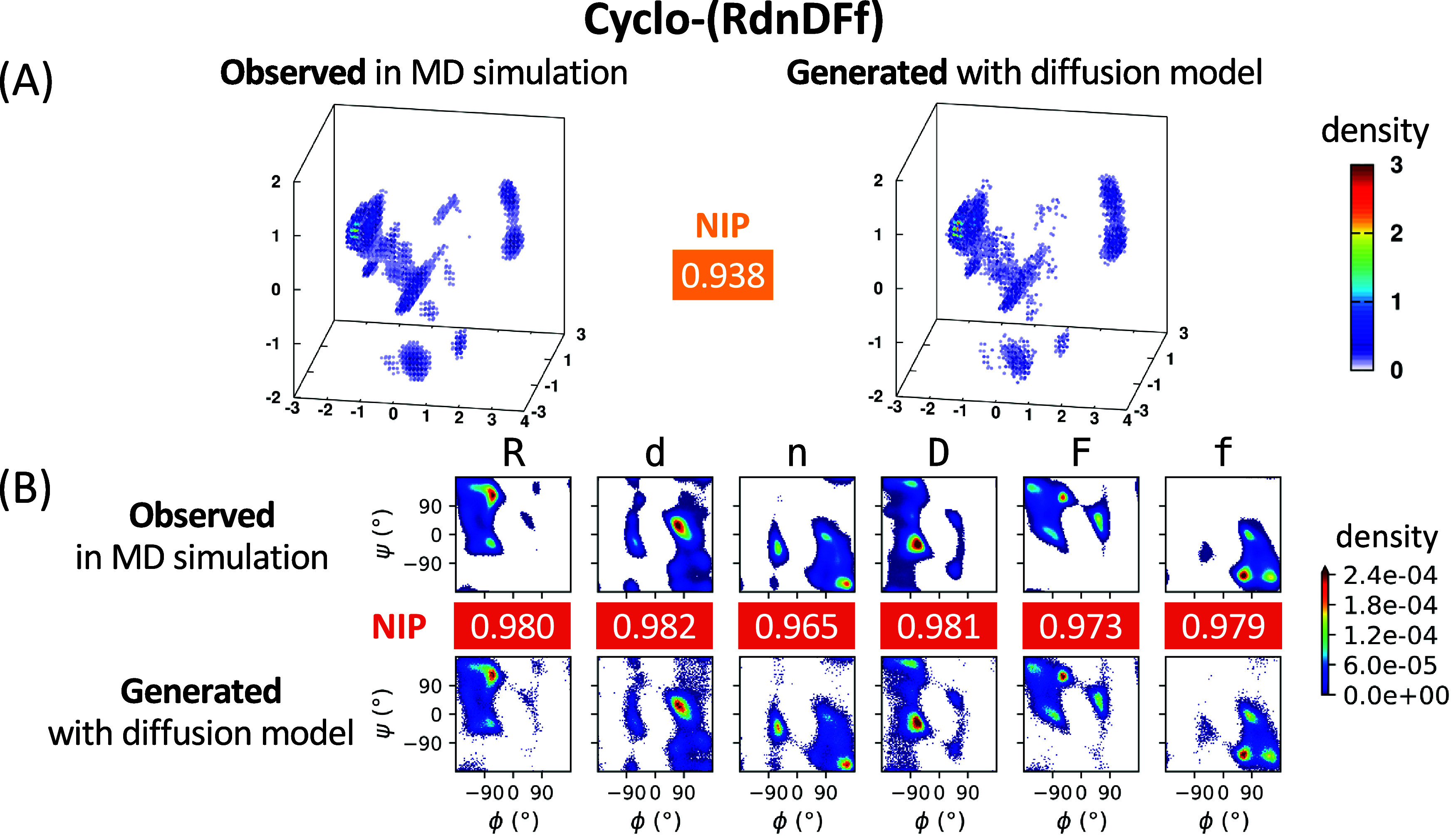
Prediction of the structural ensembles of cyclo-(RdnDFf). (A) Projections of backbone dihedral angles (ϕ, ψ), expressed as sine and cosine, onto 3D principal component spaces. NIP between the 3D probability distributions of the MD-observed and the diffusion model-generated structural ensembles was 0.938. (B) Ramachandran plots of the MD-observed and the diffusion model-generated structural ensembles. Per-residue NIP between 2D distributions of the backbone dihedral angles (ϕ, ψ) ranged from 0.965 to 0.982.

### Diffusion Model Accurately Predicts the Structural Ensembles of Cyclic Heptapeptides

Similar to our performance assessments of diffusion models for cyclic pentapeptides and cyclic hexapeptides, we generated 50k structures of each test cyclic heptapeptide with the diffusion model. We then compared these generated ensembles to those obtained from the MD simulations using NIP as well. Two complementary representations of conformational space were considered. First, we performed dPCA followed by the calculation of NIP between distributions of backbone dihedral angles projected onto the top three PCs (see Figure 6A for cyclo-(vVrdNGV) as an example). The resulting distribution of NIP values across the test set averaged 0.869, showing that the model generated structures consistent with MD ensembles (Figure 7E). Second, (ϕ, ψ) densities were used to calculate per-residue NIP. As shown in Figure 6B, cyclo-(vVrdNGV) showed highly similar (ϕ, ψ) densities between the MD-observed (Figure 6B, top) and the diffusion-generated structural ensembles (Figure 6B, bottom), as reflected in their high per-residue NIP values. Across the full test set, Ramachandran-based comparisons showed an even higher average NIP of 0.950 (Figure 7F). The discrepancy between the average NIP in PC space (0.869) and per-residue NIP (0.950) in ϕ, ψ space indicates that the diffusion model reliably captures fine-grained per-residue torsional preferences observed in MD but shows a poorer performance in capturing the conformational ensemble preference as a whole. The noticeable drop in performance, compared to the diffusion models for cyclic pentapeptides and cyclic hexapeptides, can likely be attributed to the significantly small ratio between the size of the training data sets (705, 705, 734 training sequences for cyclic pentapeptides, cyclic hexapeptides, and cyclic heptapeptides, respectively) and the size of the entire sequence space (76 thousand, 950 thousand, and 12 million total sequences for cyclic pentapeptides, cyclic hexapeptides, and cyclic heptapeptides, respectively). Furthermore, larger cyclic peptides are expected to have greater available conformational space and tend to be more structurally diverse, thus making it more challenging to describe their conformational ensembles.

**Figure 6:**
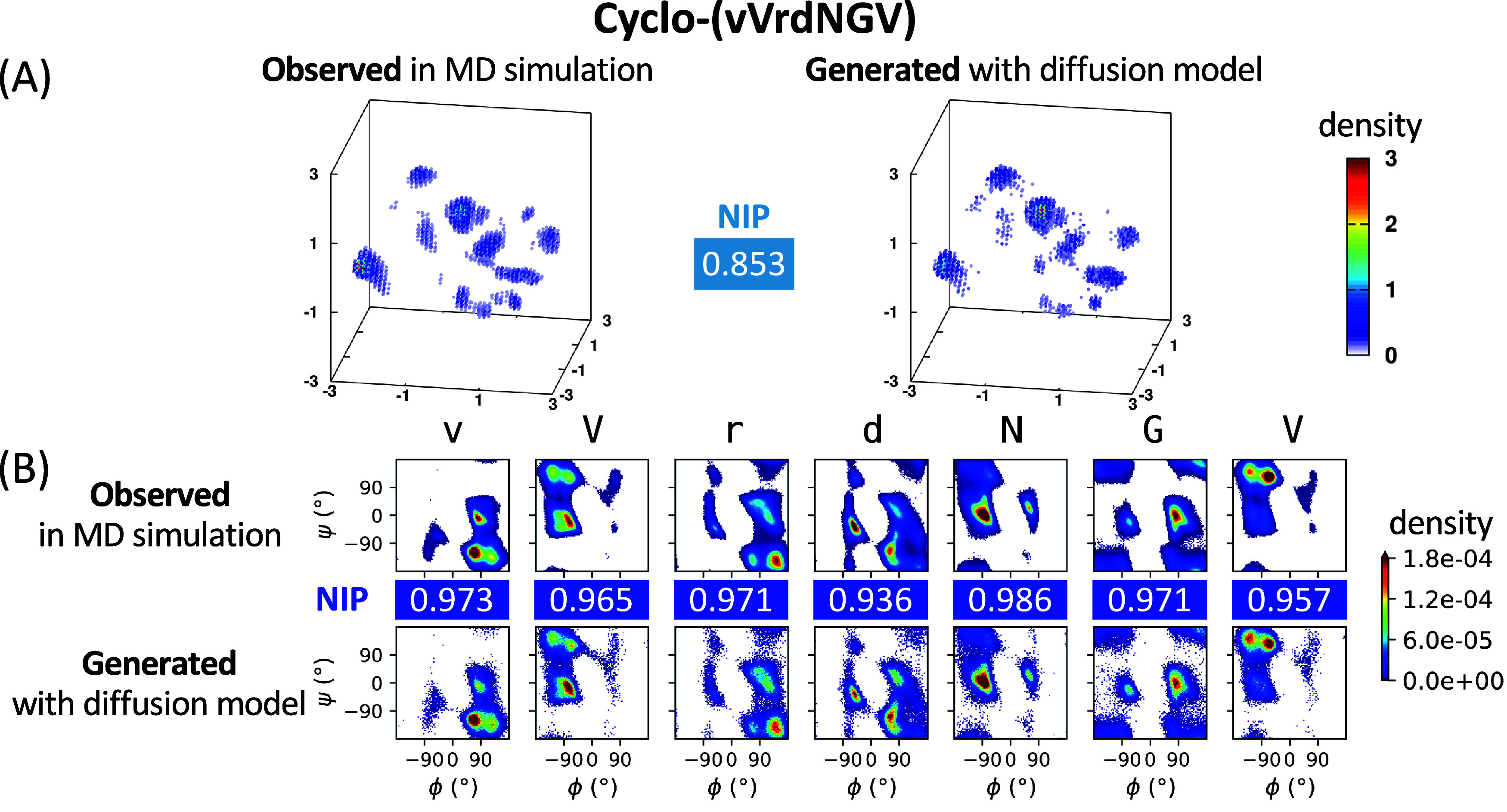
Prediction of the structural ensembles of cyclo-(vVrdNGV). (A) Projections of backbone dihedral angles (ϕ, ψ), expressed as sine and cosine, onto 3D principal component spaces. NIP between the 3D probability distributions of the MD-observed and the diffusion model-generated structural ensembles was 0.853. (B) Ramachandran plots of the MD-observed and the diffusion model-generated structural ensembles. Per-residue NIP between 2D distributions of the backbone dihedral angles (ϕ, ψ) ranged from 0.936 to 0.986.

**Figure 7:**
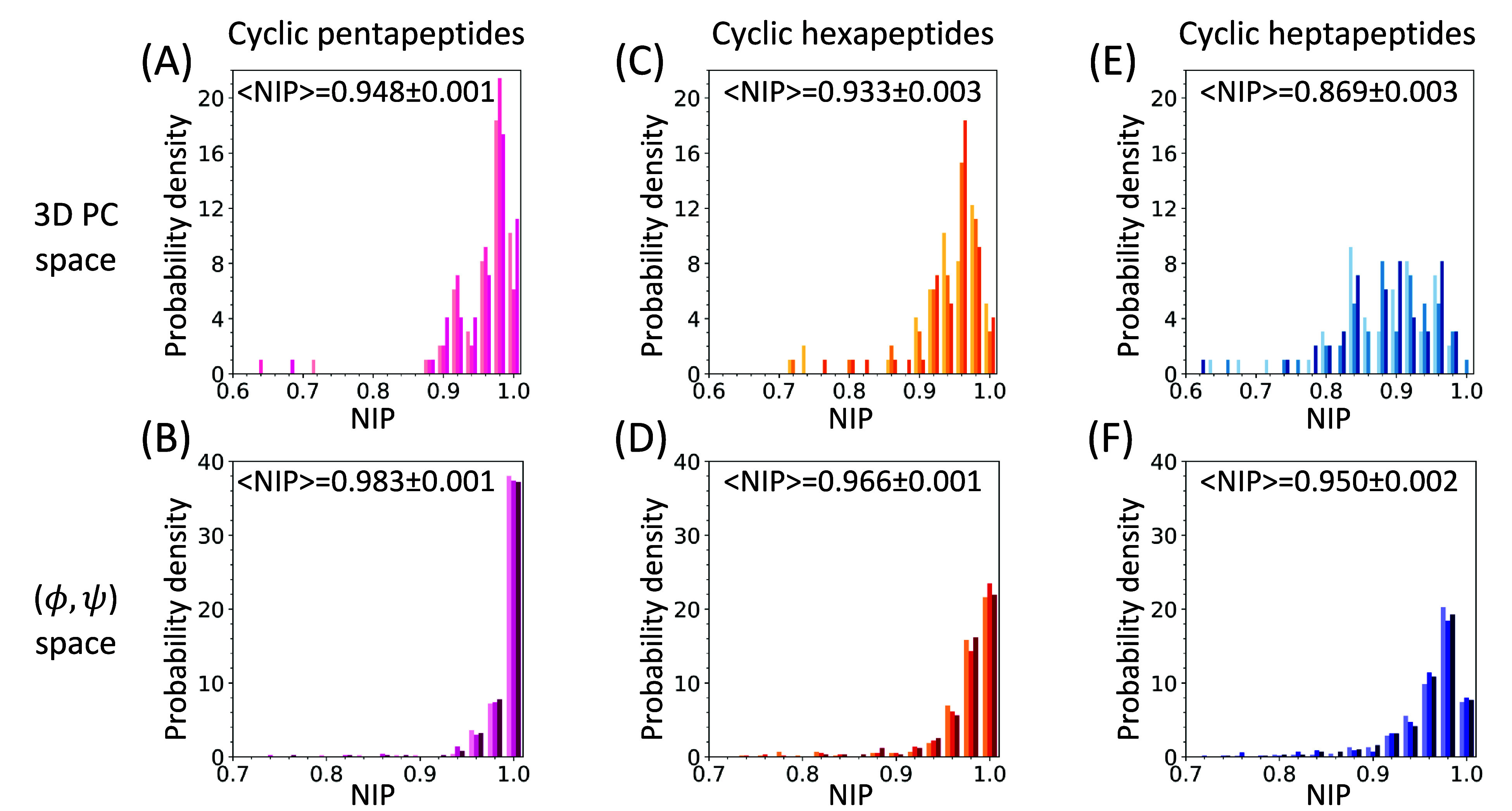
Diffusion model for cyclic heptapeptides recapitulates the MD structural ensembles less well compared to diffusion models for cyclic pentapeptides and cyclic hexapeptides. Performance of diffusion models on the test sets of (A, B) cyclic pentapeptides, (C, D) cyclic hexapeptides, and (E, F) cyclic heptapeptides. For each test sequence, 50k structures were generated with the diffusion model. Top row: distributions of the NIP values in 3D principal component space. Bottom row: distribution of the NIP values in (ϕ, ψ) space. Results reflect three independently trained models; ⟨NIP⟩ represents the mean (±standard deviation) of the per-model averages.

### Diffusion Model-Generated Backbone Dihedrals Result in Valid All-Atom 3D Structures

To generate 3D structures, cyclic peptides were first constructed in Chimera from the amino acid sequence using the backbone dihedral angles (ϕ, ψ) predicted by the diffusion model. These initial structures were subjected to energy minimization in GROMACS with harmonic restraints applied to the predicted (ϕ, ψ), followed by a second unconstrained energy minimization. Figure S7 shows the resulting Ramachandran plots. Figure S8 shows the NIP comparisons between dihedrals obtained from MD simulations, those generated by the diffusion model, and those extracted from the generated all-atom 3D structures. In most cases, the generated 3D structures preserve the diffusion model-generated backbone distributions, with PC NIP values typically ≥0.9 relative to the predicted (ϕ, ψ) angles. Deviations primarily arise from the cyclic closure constraint. The diffusion model does not enforce loop closure explicitly, but the training set of cyclic peptides enables implicit learning of (ϕ, ψ) combinations capable of forming a closed backbone. However, we observed that some generated (ϕ, ψ) angles cannot cyclize without deviating from the originally predicted values.

## Conclusions

Current computational methods to predict cyclic peptide structures are limited to the prediction of (a) homochiral cyclic peptides due to the training data set bias or (b) low-energy backbone conformations requiring subsequent sequence design. Because cyclic peptides tend to adopt multiple conformations in solution and rarely populate a single conformation, the ability to predict their structural ensembles can greatly aid the de novo design of cyclic peptides. Notably, StrEAMM models can predict structural ensembles of cyclic pentapeptides and cyclic hexapeptides but require a scaffold-specific structural digit map to represent structures.

In this work, we introduce an approach to train diffusion models directly on MD simulation data by representing the structures of cyclic peptides in terms of their backbone dihedral angles (ϕ, ψ). The trained diffusion models can accurately predict structural ensembles of heterochiral cyclic peptides observed in the MD simulations. Additionally, all-atom 3D structures of cyclic peptides constructed from diffusion model-generated (ϕ, ψ) angles faithfully reproduce the predicted (ϕ, ψ) distributions.

Several further advancements can be made to improve and generalize the diffusion models. For example, additional training data and a comprehensive hyperparameter search can likely further increase the performance of the diffusion models. While the current diffusion models use simple one-hot encodings, we plan to incorporate more descriptive features to represent amino acids, such as Morgan fingerprints^52^ and MACCS keys,^53^ to improve the generalizability of the diffusion models to new amino acids. Notably, MD-derived features have been shown to outperform other amino acid encodings.^54^ Hence, we hypothesize that the incorporation of MD-derived features will allow the diffusion models to generalize to new amino acids, both canonical and noncanonical. We expect the diffusion models to serve as powerful tools to enable efficient and accurate prediction of structural ensembles of cyclic peptides that can substantially facilitate the design and development of cyclic peptides.

## Supplementary Material



## Data Availability

The diffusion models and MD simulation data used to train these models are under a patent application (please see Competing Interests section); nonprofit and commercial users interested should contact the Office of Technology Transfer and Industry Collaboration at Tufts University.

## Notes

Lists of sequences in the training and test data sets; data set splits for hyperparameter tuning and model training; size, performance, and computational cost of models used in hyperparameter tuning; final model selection; parameter sweep over the guidance strength; wall-clock times for MD simulations, model training, and inference; comparison of cyclically permutated sequences; analysis of generated 3D structures (PDF)
