# Supplementary material for: Fast Generation of Simulation-Quality Structural Ensembles of Mixed-Chirality Cyclic Peptides via Diffusion Models
Source: J Chem Theory Comput. 2026 Mar 12;22(6):3103–13. doi: 10.1021/acs.jctc.5c01862 (PMC13262345; doi:10.1021/acs.jctc.5c01862)
Supplement: Supplementary file 1 [file ct5c01862_si_001.pdf]

Supplementary Information

Fast Generation of Simulation-Quality Structural Ensembles of Mixed-Chirality Cyclic Peptides via Diffusion Models

Nomindari Bayaraa, Maxim Secor, Marc L. Descoteaux, and Yu-Shan Lin*

Department of Chemistry, Tufts University, Medford, Massachusetts, 02155, United States

*To whom correspondence may be addressed. Email: yu-shan.lin@tufts.edu

List S1: 502 semirandom cyclic heptapeptide sequences. Sequences whose simulation did not reach convergence after 400-ns are colored in red.

NanSrSR	avAArav	dSAFRrA	VvNNRFN	fvafDNF	aDarAAn	NsArSds	FnfFaGr	vAGNFss	DAFaFsS
rnSFfnV	ffvnrDR	frRNFDF	RRNDSDS	sDaVRsS	frdVfDr	fRGGRsf	dNdFFvS	nDvVafn	rVvGrGD
afffsAs	rRsnnfS	DRFVvvf	vGAnrGN	vARAddd	DGVvaDN	adADFnG	vfaSRrF	AvrnGvr	FvnvAdD
dnSnfVf	AAAFfDd	rRFvNVG	fArFFVN	rsfsnSa	VVnavGR	vvSvvdA	GnsfVDf	FRFnVFs	rvnDSGA
RSsRNrA	AsndrNG	DandRdG	fdFvaAn	VnssnGF	rrfSaDR	DnDaASf	dVrNVSV	FAFADds	aSrdfAv
rGFvdnf	dVsvRFR	fVraDDV	NFANVav	AnARnSv	DradSNS	nrNdrAr	DfSSFVF	vrGrSSa	vArVnfD
vaVAaSs	DSfGDnG	FvDvnfs	narGRnR	AVRfVAN	RFGRDns	vRVFvRn	RRssSdF	sdGADGr	aavDSNR
NAGRrDN	rNrnVRF	vANDDDr	GvVvDrG	vFAvFNd	fDGfVGG	DrSfSRA	SGDfsSv	vNnsFnv	ddnNGDN
NdVGfSf	AGAVdnA	VVDvvVG	DaDVDVA	rsnaSFF	asSFNfd	SaaGsnN	RAGaRNV	ARNfass	vnNnarS
rVDRGNs	rFaNnnn	VArRraA	ndDsrSD	SnsaaVV	DRNnSSD	RRrGana	NnDnFVd	DVrFGSF	faArNNR
naAsDdn	DAAfvvG	dARGssF	fFRANsv	NGVaddV	FSSNnfA	nsdNFvv	vVSanVA	dsVvVnN	aGSasFV
NaNRNGn	fDVGRaD	DDFFdrs	asdDAGs	vfVRDDN	ADVFGvN	rnaNaFa	AGfsVsV	nRdsnrf	afNvRvN
GvsnfvR	VrdNVnV	nvVrSaV	DFsGGaS	rFVSRfv	fGGnrSs	saAFrdF	DnnSDsa	rrFAffF	FsDrAaG
vfNDsRV	VfFDAfA	aNVDdFf	dDDvNDr	NsSRnGR	nFNRVsR	AdarVSF	dNvsdVA	aVvdGdr	GvRdvvr
fndaRSG	VNDFraS	aNdfrrR	fNAAdvG	fSFaaNN	FvFfRNA	ddDNAsR	AaRGAdF	AGvaraf	GfvddVR
dSnAaNG	vGVRdRs	RFSFdFS	nAvAfSn	SGNGaDs	dndAGGF	fvGafav	nnVvnAF	dSDRVDD	dNARRva
AfaFnaG	GnfnDVV	afRDVvR	VdrVASv	vDdVdGS	vGFARfF	aGRRRDR	NAvfdfF	SAsVGFf	fNFFSfn
dFrnDAR	DRvGSfs	dsDFSNV	vdDrRdD	NRaFrvG	rFNDvrS	NGFRSNs	SAGDFAG	NAadrnn	FGAGnGn
DAvGsRv	RnsnAGd	dRrRRFd	dAnDGDs	srnfRrs	rVFASNG	Nvndnva	VnGsNDA	SSANfRa	dvNsfFV
dnasDsN	sDNRDsn	FnsVrVN	NNGsvFn	ADDsvDn	rfNssRG	vfARVSf	vSRvdfs	nSRSDav	dRNRrfd
nVSGddG	aRsdrvr	SSRsDGv	NsRDGnd	FdSvffr	sSArrdn	SVSdRvf	GdnrRGR	RRGnnRf	VdDdRRa
rrAGFGs	sdvVFrV	RDNffdv	DnRNdaF	RvrsVVA	RvSadfa	GSSfRAn	RnfGsAN	DnffAnN	AarrGff
DGsVffa	nFDdDSS	AFsAVsG	nRGSrfF	dVDANNS	VssdanG	dDRnNvF	GvdVSSv	rasfRfG	ASSVDGR
ranNrDn	GGGDvaF	dFdVVVd	ndvsDfr	SsGvGGV	SSrnsvd	rsAFGnD	dvaaFSD	fdGfFda	sNRsVVf
NvasRsN	FFGfdVF	ANasaSN	SsnRFFn	aNfrDss	DvRrNFr	AVNsaFV	dDVdRRf	DDGGsdn	rRfrSFV
GdvfraG	nGARrdv	SrASAvs	fanFRvs	AASRGFF	vrfRdSF	SdfVVsf	avVNAds	ADnvsvs	rfAAVSd
ArsvrDD	VfRvnRR	vfnSGaG	SvRRdfD	sSaGFrG	GNSNNFd	aFvsRAS	vfGaFAn	nSdASrG	rNRAfnr
RGVGsfN	DSAnRsF	SRdrrnR	FdsdFVD	DvsVSAa	rDGardd	DADNdGs	NdNRSnG	FGrDrrD	fGVAADf
FNrFsNV	fSDfNGR	rsGVrAN	daANnvr	RSfdRGD	ADAaVfr	FrFSGrn	srvRfAf	VFdfdrG	DaSaanR
rarFfds	asNfVda	vDNSssD	FVfvDFN	aaAvnVf	faGaVsr	adGavsF	nFdRfsG	VAsrAsS	DFDnSNf

SVVRrnv	fnNRAvD	aNSvrVV	fNfFsVF	VNNnFvV	dGvnafG	NFSrVdA	nNVfSsN	nDdrdAa	fSNdSdd
nRVNdRV	adRDFVs	nDfvVRR	rNSAAaa	drSAfNr	ssfvSfA	RvFSASG	sGaARad	AFDaRAr	VNnGGNN
SFnDFav	vAaAGrN	aVGNafs	vSARsGr	NVrGASD	GANdAFV	DSVfNVs	dsfdArG	rnAsGFn	rFrAfVs
DsASFsn	aDFRfna	NASVsFG	NsdSaNr	AVanvnF	DasrNad	NNDRAaF	NnVNSnD	VDSFGdA	frsRGaa
RdnRvva	FfGRdAs	GNVAnns	fVnSVrf	FVVFSnv	FRndFDr	vvAFNnr	vDsVRSr	AfRSVRa	VNVVsAn
SvFasnF	nrdGFaR	DGAvdrF	sSSSdnV	VnnGrdS	DDafVNv	sDVsnVD	RDASsar	RVRNNdS	FvASdDv
DsGSnSA	FRNvVDa	SAdAdrR	arfssGf	rsNFFaV	SGnannd	VNRdaGD	AdGGAas	dRarvFs	SDVRVdV
fNaVNaR	SaAdRSF	sAGVfGS	GfrVfAN	sSNvSnr	GRFaDSd	afvsGRS	aVasGDd	SRDrNfN	aFddssN
GSdDGSG	VFVRnVa	rNArDVS	DsFSVvF	FnAnSfV	FNaAafF	dDfFGDG	afSvanf	NvNRvVs	aNDNrGd
RAAvSNr	GFSdvSD	avvRSvd	NavdRFD	sVDnVGa	FFRsAav	ansrsaR	GNraNAf	NSVFanA	FNsFNfG
sFrSGsG	AdnGfRn	AnsAAsN	fFAsvVA	ffnFfsd	GVnrVaS	FFDDSrs	RSSsdsR	nGdFndf	fdSnVnA
DRdNSGF	VaDdNrf	aSffNNN	adnFaSd	adFNGvD	VdFGaNs	rDSndVa	rDaNFNS	SNFRDfV	GGrAnFR
dsvvDVn	daSnNsG	dGrrsrD	FFsrRVA	dVvADvG	AvNrVRv	SfaVSvN	RSdSsFs	NvvvFGF	GNnRava
DnNfvNd	vrRnnvd	fSGSNDf	vRsrdsA	nrAVvsa	GdNGNDa	dSfrNDd	nvGnvNf	fASnRAs	RrVGDDn
DfRsRfD	RasvNGS	DAdfRVr	vsNSSGv	SAVrraa	fsRaRDv	FArdRAR	SsVnDRD	vAsAfrf	aSvsrVS
GnRrSNa	fNndGVd	NsnvDGN	aFfVvSr	NrSVaGV	RFsfrvf	AsddAfD	ADRsaGf	vFrfGfG	nASDNGG
sNrvVfn	DVaaadN	DnaVDNs	SFrRvRN	nFsvnnF	aRVaADS	FffDaaR	dFaAANv	fFSsSfF	rvaFFDv
nddNaDA	nNFGVDr	fDfdNfs	VVvrNnA	GAARDdf	SVGvAVG	AVFRavR	VrvNvrr	nadvdsS	NaasssV
GSDvSsr	sVaRrvs	nFFfAdV	fvFRVGd	NNrrrvd	RAFdvDR	VdSrrSn	fDsfnAr	fadaDrD	FdNRRna
GNfNFaf	adVaFdd	SFRRsRr	NarSDfA	daRddDF	vffvNVF	NFfRVFF	rDVRVDa	vfnGDDd	DDrDaAR
nrvdNvS	fddAaGN								

List S2: 300 random cyclic heptapeptide sequences. Sequences whose simulation did not reach convergence after 400-ns are colored in red.

ASvSsda	dfvNsRG	VdRSDnV	GGDvvVf	NVvFsdR	ADNAFnN	sdFarRG	rAvnrGa	RGNVNRr	nsDRDna
NFvfDnr	SDANVfn	SAnvaav	VvVaffs	GfdaNAv	VaRdNNV	dRVSaNa	vsnGvGn	sDsDfaF	NNvGfDd
NAAVnNR	GAsFrrs	SFdsDva	FvNdann	GvassRr	sfAfFav	nnSnfAD	FnfFSDv	dnGfAGa	AaGnffa
vAvSAGf	DSGGddN	RVVndrS	FDAsfDV	AfnfavF	GfsfDDs	NfrSnFN	fnDrAda	dsFvsdv	NADFVRR
fFsafAv	anGGfFF	sGfvNsA	fSRFsGv	rsrGGvN	snAFGvN	nnsasfD	sFsDrsS	VVvnNFV	FGNNdDA
rVrsvvV	nNaVVND	aSNnNGD	AVSsGnN	AfrFNfF	rGafrsf	naFSSsa	rvvnfDs	RNAnvdA	RNnVVAf
AvGVnRR	rNARVdF	nvaSrdV	fRSDSFF	GnfvSvN	GfFfffs	GGnVVGF	GnNFFFa	nvddVFA	dsVAGRV
NraANfG	SGDfFVR	sfFNaaV	nDvNNfD	aDRrnnf	RGVvSRd	RnSGFAA	dGfVSns	rNvSrff	DrVGGaF
nFvVrdR	sRVVNRn	NnfNSrN	DVNSNNv	RaFrFrN	SGndfda	RFnFDrN	RVsnsvS	RFFvvrA	DsVnaSd
DsssVVR	DsdAdDv	dGNsGaa	svDRDNs	RGrAnRV	nnASrDV	afADGSv	ranSnVa	fsDvaNV	DnNrVSa
RAVARsR	dRNffAV	fDGASdF	FDvvDAa	NSvfSvA	fdAsDDS	VNRFvNn	rdFFsVR	RSvadFD	sNsDSnD
GrRRGAS	sDVdRfS	sDvadsG	dAsDdGF	GnVAVfv	RfFGNaR	nVfFSra	VsvnfaG	RfDRdSA	nAdSsRr
VdaVrNf	FavnDNR	NdvVVaS	nGnvADv	GrGnArn	NAFRann	FfrrnVR	FafaFVf	SFrGDfr	dddNAsG
VfNRnVs	ffnSSRS	sGNanfR	advnSDR	aRssSsD	NAnvRGD	DvRraGa	aNFAARd	DSGVnRS	aDdsFFR
fravdAV	DRFanvR	dSDnFfV	VRvSGSr	nfSrVnV	SvradsG	aGnsrFa	NrRnVvG	ASGvDAV	afNArFR
nSsGvRA	sRasssd	NDdsVfV	GDnNRAV	AfsDdfS	SFSVVAF	GFdfsAV	AnarNrS	AASFADS	nNRVSfV

dNsDDNV	GVNffaV	nvsndRD	SDSvnna	DfvDdrV	VDvaADA	GRsndvd	DSnFdDs	DDvanrd	vSFnNAA
asrVdVD	vSNaaFr	fsFRvas	SfGsdsr	dfrAsvD	VAVRGNG	srsaAAV	faRvAaF	FSaRDdD	FvdNGna
nVSnDdf	dGFfSVs	RFFvdnN	AdDRRFa	GfsSssA	srGdNDD	DNNrVna	RsAFRDN	GnrANVG	sranRra
aRVsnvD	aGSfDaf	vrNrvaf	rSfnSvG	nfVdvfa	FrDNDar	nNArrRr	adrFSVF	RNFDfGa	aFAArnv
NsvRasn	dGfDDVV	sfsDGds	VSnfAvN	AvAfNRS	FsGNfvS	rrnNNaF	nRDNAvV	FVrnARv	sfDdsNR
VnVDASn	dNGsAAN	aafvSAA	GAVvvfd	fGGDNSV	vFnSFfS	RdvvGNN	SVaaNSD	sVDGvdS	advDvDG
SNAdnvv	VsFrFfG	SRFVVRF	NrFavsf	sDannRd	sSrVDVs	RSFVfAr	nDvAsVs	rGARSfn	GsVRNAs
NVGsFVG	rGNNFGN	nFRrSAr	DfGavFd	NffASFr	RfddNRA	VnDRrnD	sdvAnad	Nsndnrr	FGfRvrG
sNfDSfV	vvfGnnr	RdVRRRG	AvnrDGR	GGvFsAF	DRfGfRF	AfaavSR	FAVAanR	aGDfVFG	RsVGrfA
VdGFava	saVvRfd	RFAvsNN	nNdARNF	nddVDnD	ASFdAdd	fsrGNDn	DGArNnd	AfDdVvR	DNnsVrf
dDGrAfN	nfRNSda	fRNARSa	NDnaarn	VRfVRAD	FvNdAfN	ravvsGR	fANSRfN	dnvaDfV	rFAsRSa
FnGsVvN	dAAfrNG	sSGVrVF	dRffFNn	VFdDfGR	nrrSSaG	DnvSvnv	VGDFNds	GvsASFR	drrdDfD
dDdnDDf	RAdSvfN	SVFVsaR	asFdsNG	GssaaSn	GAArvNa	GRFGRrV	DsSvdVR	rRfnRFv	FGNRRsA
SVNSAra	vrNnfGn	sarsvVv	naSNDGV	RSvaGns	SfNGGNd	naGAFvN	sGrvNrR	nARsAdr	RFFvAAd

List S3: 53 random cyclic heptapeptide sequences. Sequences whose simulation did not reach convergence after 400-ns are colored in red.

sfvVVGR	anSnNnA	AVDRfsV	DSAVaAA	NNdsNsS	fGGnfaF	dvVaAAD	rVsGnrG	dAaVDsn	AFdfFNG
vVrdNGV	vVddADD	nNsfNDn	Rdavdfa	nvAVfrs	VGrvaDS	vGSFanf	vrAFDaS	GavadAF	ffAfARA
frGrDsA	SsSRdaD	dNNGVVN	RvavfSR	SfGFDfD	AAsdDFd	RrAFAFA	NGRNNSV	VvdSrvn	NafsfDv
DVADdFV	NAAsDAn	raGsVVs	adDvDfn	vRDGaFD	anSarAD	rGNDrff	nGrNSrv	VvfGAVS	rFvFdas
RnfDSVv	daFvGna	dNGNRsD	nNSrSNN	rnAFfrD	AFDRnFV	SrrrGVa	faSsARG	AAVnVDG	FSnAGAr
vsSrFaa	vFRavNN	GDfDafn							

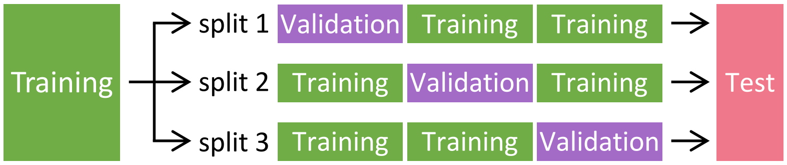

Figure S1. For all three systems (cyclic pentapeptides, cyclic hexapeptides, and cyclic heptapeptides), the training dataset was split into a base and an auxiliary training dataset. The auxiliary dataset contained 150 sequences of cyclic peptides, which was then split into disjoint cross-validation folds of training (green) and validation (purple) sequences containing 100 and 50 sequences, respectively. The resulting three models trained on three different training/validation splits were used on the same test set (pink).

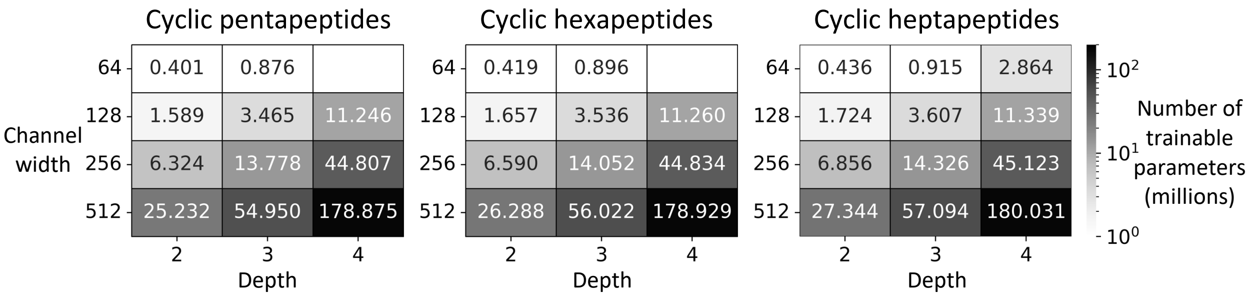

Figure S2. During the hyperparameter tuning process, the channel width and the model depth were varied. The resulting models had a total number of trainable parameters ranging from 401 thousand to 180 million.

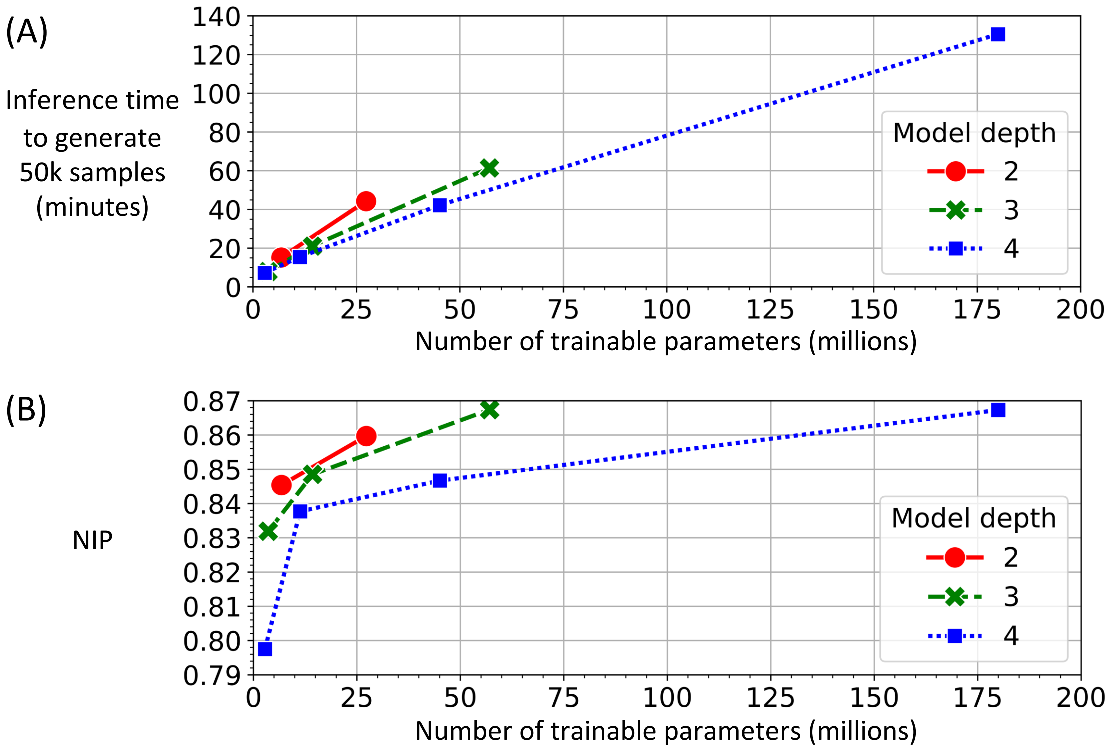

Figure S3. The comparison of diffusion models used in hyperparameter tuning. (A) The time needed to generate 50k samples increases linearly with the number of trainable parameters (model size) on a single NVIDIA L40 GPU. (B) The performance of diffusion models reaches a plateau with a larger model size. (A) and (B) are based on the hyperparameter tuning models for cyclic heptapeptide with a learning rate of 1.0×10^{-4}.

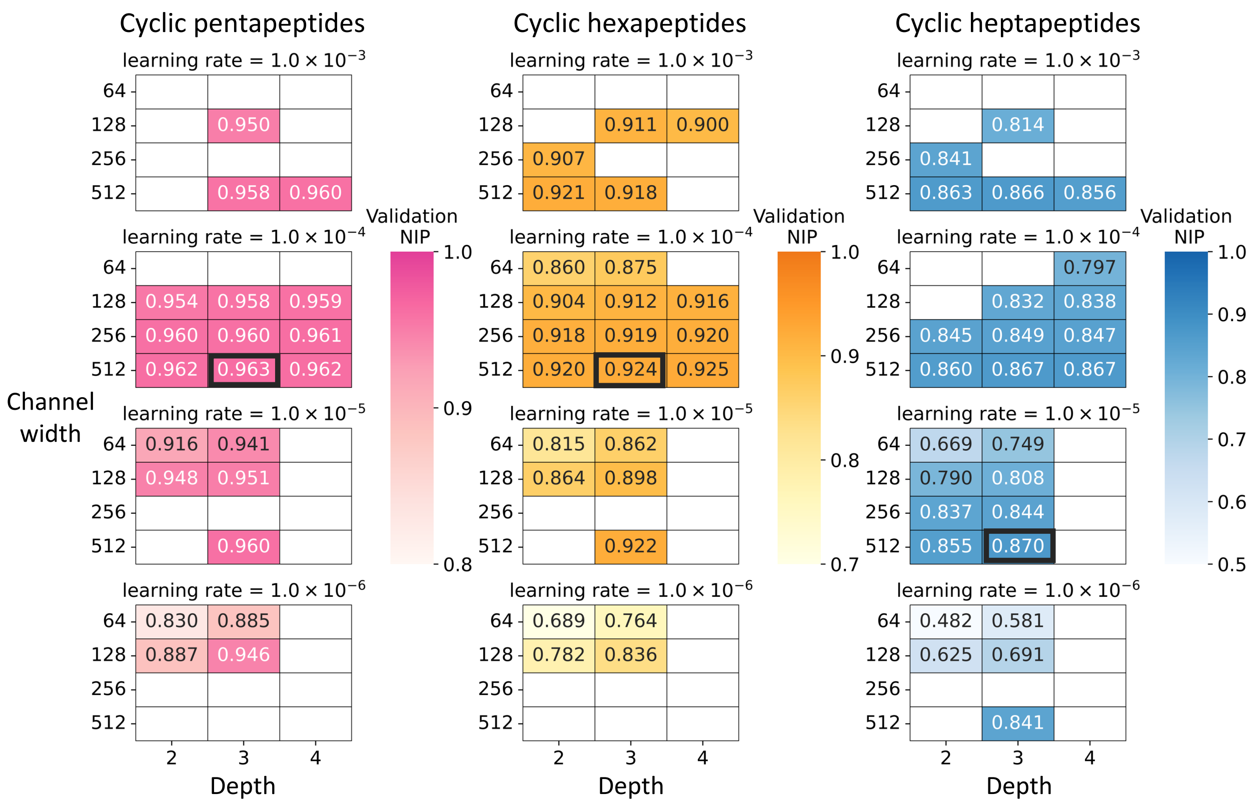

Figure S4. Hyperparameter tuning. We varied the learning rate, U-Net channel width (number of features), and U-Net depth (number of down-sampling layers), resulting in a total of 12 models at each learning rate, with the total trainable parameters ranging from 401 thousand to 180 million. The final models used on the test sets are in black boxes.

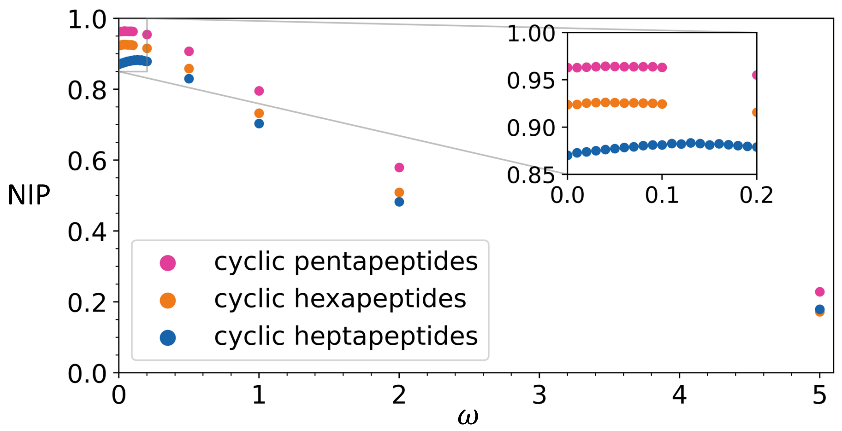

Figure S5. For each diffusion model, several ω values (0.01, 0.02, 0.05, 0.1, 0.2, 0.5, 1.0, 2.0, and 5.0) were initially used to infer validation set sequences to determine the impact of ω on the model performance. Then, the additional increments of 0.01 were used to get a refined view for small ω. Overall, increasing ω leads to a performance decrease, but a slight performance improvement can be obtained when ω increases within the range of 0–0.2.

Table S1. Wall-clock times (in hours) for molecular dynamics (MD) simulations, training and inference times for the diffusion models. MD simulations for cyclic pentapeptides were performed with 15 Intel Xeon Gold 6346 CPUs and a single NVIDIA Tesla T4 GPU; MD simulations for cyclic hexapeptides were performed with 17 Intel Xeon Gold 6346 CPUs and two NVIDIA Tesla T4 GPUs; MD simulations for cyclic heptapeptides were performed with 19 Intel Xeon Gold 6346 CPUs and two NVIDIA Tesla T4 GPUs. Diffusion model training was performed using 32 Intel Xeon Gold 6448Y CPUs and eight NVIDIA L40 GPUs, and inference was carried out on four Intel Xeon Gold 6448Y CPUs with a single NVIDIA L40 GPU.

	Cyclic pentapeptides	Cyclic hexapeptides	Cyclic heptapeptides
100ns MD simulation (single sequence)	53.86 ± 1.93	30.92 ± 2.55	33.11 ± 1.58
Training time (final diffusion model)	43.10 ± 0.10	69.65 ± 0.31	85.52 ± 0.31
Inference time (50k samples)	0.81 ± 0.01	0.91 ± 0.01	1.00 ± 0.01

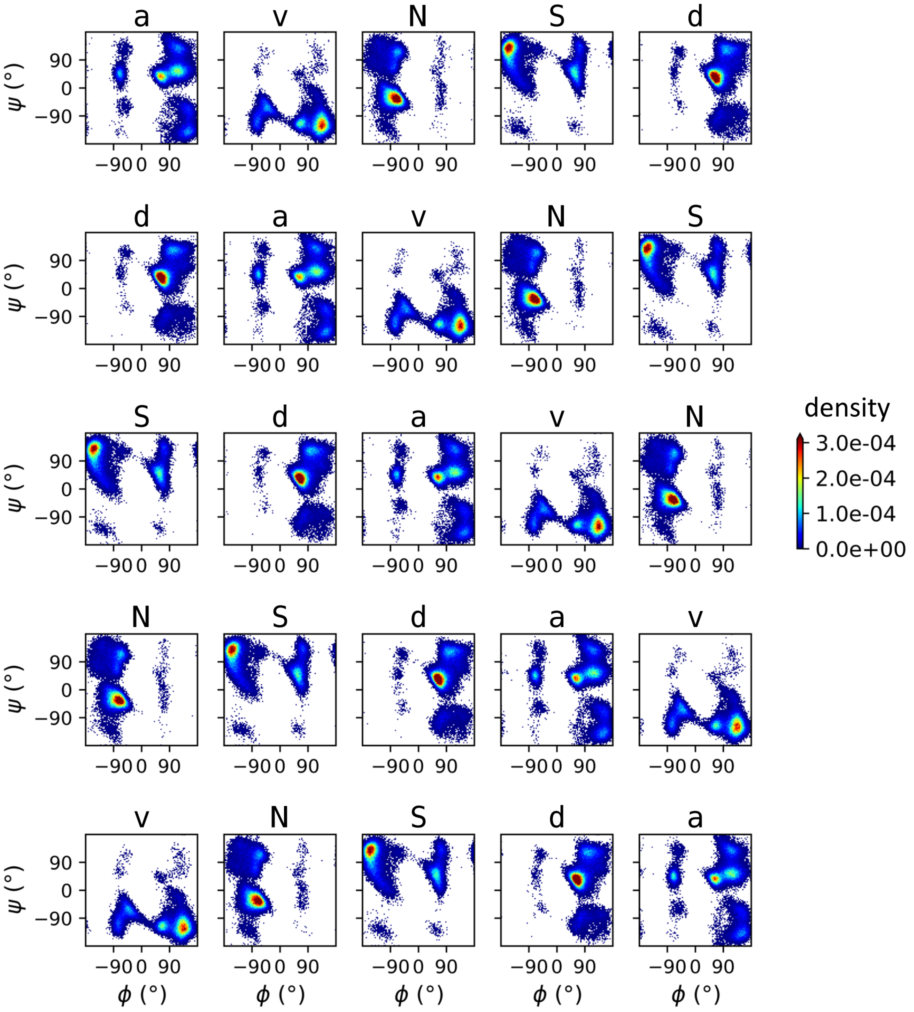

Figure S6. Generation of all cyclic permutations of cyclo-(avNSd). Cyclic permutation equivariance is ensured by augmenting the training dataset with all cyclic permutations of each sequence. As a result, when 50k samples are generated for cyclic permutations of a test sequence cyclo-(avNSd) using the diffusion model, the resulting backbone dihedral distributions are properly cyclically permutated.

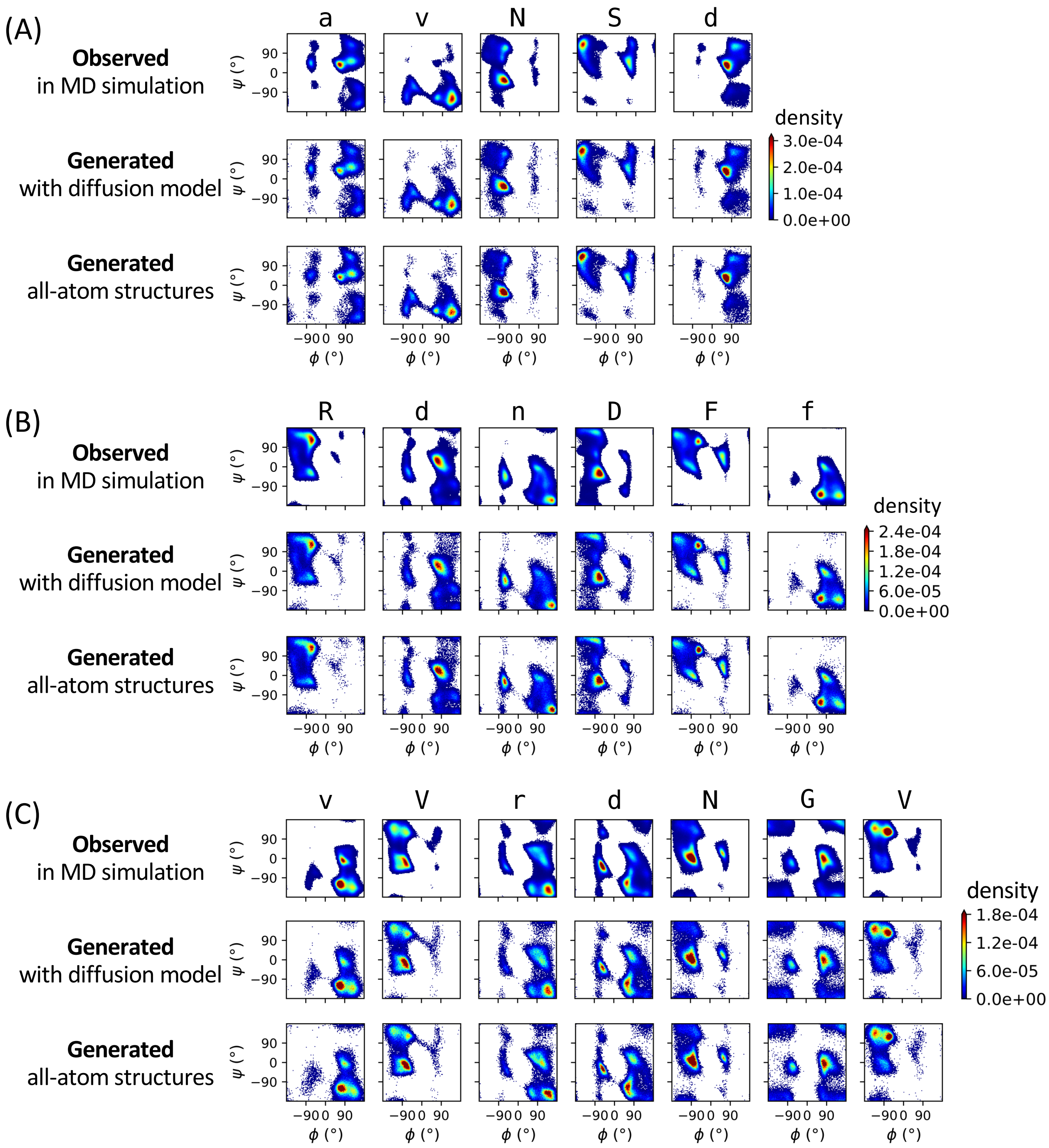

Figure S7. Distributions of backbone dihedral angles of 3D structures constructed using the diffusion model-generated (ϕ, ψ) angles for (A) cyclo-(avNSd), (B) cyclo-(RdnDFf), and (C) cyclo-(vVrdNGV). All-atom 3D structures of cyclic peptides were generated by first building cyclic peptides from a given sequence using the diffusion model-generated (ϕ, ψ) angles. Energy minimization was then performed with (ϕ, ψ) constraints, followed by a second unconstrained energy minimization.

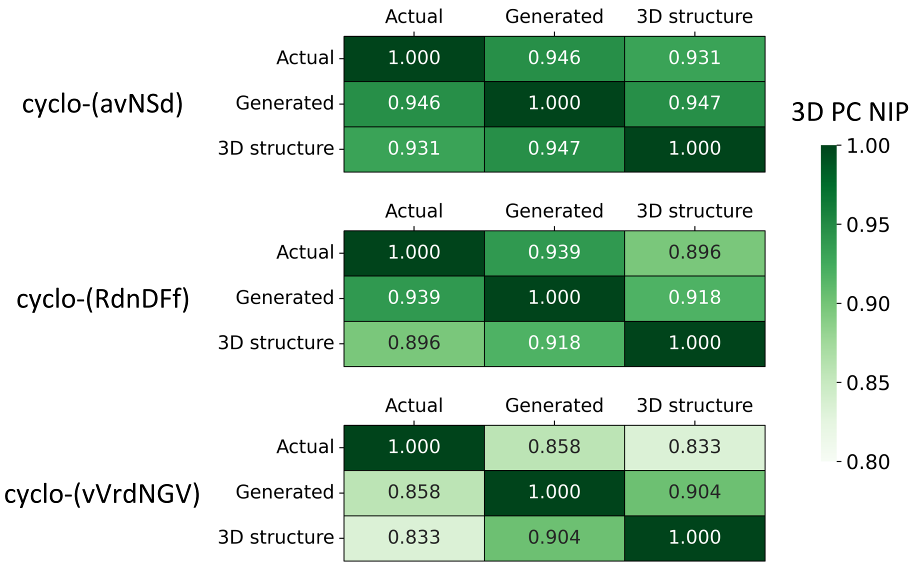

Figure S8. NIP in 3D PC space between backbone dihedrals obtained from MD simulations, those generated by the diffusion model, and those extracted from the generated all-atom 3D structures.
